# Early behavioural facilitation by temporal expectations in complex visual-motor sequences

**DOI:** 10.1016/j.jphysparis.2017.03.003

**Published:** 2016-11

**Authors:** Simone G. Heideman, Freek van Ede, Anna C. Nobre

**Affiliations:** Oxford Centre for Human Brain Activity, Department of Psychiatry, Warneford Hospital, Oxford OX3 7JX, UK; Brain and Cognition Lab, Department of Experimental Psychology, Tinbergen Building, 9 South Parks Road, Oxford OX1 3UD, UK

**Keywords:** Serial reaction time task, Sequential learning, Temporal orienting, Spatial orienting, Attention, Expectation, FMRI, functional magnetic resonance imaging, MEG, magnetoencephalography, PC, percentage correct, RSI, response-to-stimulus interval, RT, reaction time, SOA, stimulus-onset asynchrony, SRT, serial reaction time

## Abstract

In daily life, temporal expectations may derive from incidental learning of recurring patterns of intervals. We investigated the incidental acquisition and utilisation of combined temporal-ordinal (spatial/effector) structure in complex visual-motor sequences using a modified version of a serial reaction time (SRT) task. In this task, not only the series of targets/responses, but also the series of intervals between subsequent targets was repeated across multiple presentations of the same sequence. Each participant completed three sessions. In the first session, only the repeating sequence was presented. During the second and third session, occasional probe blocks were presented, where a new (unlearned) spatial-temporal sequence was introduced. We first confirm that participants not only got faster over time, but that they were slower and less accurate during probe blocks, indicating that they incidentally learned the sequence structure. Having established a robust behavioural benefit induced by the repeating spatial-temporal sequence, we next addressed our central hypothesis that implicit temporal orienting (evoked by the learned temporal structure) would have the largest influence on performance for targets following short (as opposed to longer) intervals between temporally structured sequence elements, paralleling classical observations in tasks using explicit temporal cues. We found that indeed, reaction time differences between new and repeated sequences were largest for the short interval, compared to the medium and long intervals, and that this was the case, even when comparing late blocks (where the repeated sequence had been incidentally learned), to early blocks (where this sequence was still unfamiliar). We conclude that incidentally acquired temporal expectations that follow a sequential structure can have a robust facilitatory influence on visually-guided behavioural responses and that, like more explicit forms of temporal orienting, this effect is most pronounced for sequence elements that are expected at short inter-element intervals.

## Highlights

• We show incidental spatial-temporal sequence learning in an adapted SRT task.
• Incidentally acquired temporal expectations have the largest effect for short intervals.
• The facilitation for short intervals mirrors explicit temporal orienting results.

## Graphical abstract

**Figure:**
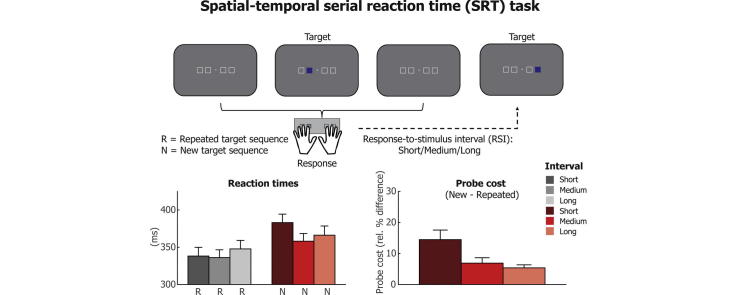

## 1 Introduction

A lot of our behaviour entails complex patterns that unfold with characteristic temporal profiles. Examples of this can be found in speech, playing musical instruments or performing sports. Timing related to such non-isochronous sequential movement patterns is often acquired in an incidental manner, over long periods of time. In this study, we looked at the acquisition and utilisation of spatial-temporal structure in complex visual-motor sequences, in which the spatial and temporal structure of visual sequences are incidentally acquired and integrated over time, in order to guide adaptive behaviour.

Serial reaction-time (SRT) tasks are often used to investigate sequence learning and memory. In classic SRT tasks (see Nissen and Bullemer, 1987, for a first description of the task), participants have to follow the order of targets presented at four different locations on the screen by pressing the corresponding button whenever a target is presented. The button press either triggers the presentation of the next target, or alternatively, a fixed stimulus onset asynchrony (SOA) is used. Unknown to participants, the targets follow a repeating sequence, usually of length 8–12. In such tasks participants generally get faster over the course of the experiment, while it is unknown to them that they learned something. When ‘probe blocks’—blocks containing a random or novel sequence—are presented, reaction times are much slower, indicating that these effects are caused by the incidental learning of the sequence, instead of a general effect of training.

Sequence-learning paradigms have been used to investigate not only the acquisition of ordinal visual-motor sequences, but also how temporal aspects of such sequences are acquired (Buchner and Steffens, 2001, Gobel et al., 2011, Karabanov and Ullen, 2008, Kornysheva et al., 2013, O’Reilly et al., 2008, Salidis, 2001, Sanchez et al., 2015, Schultz et al., 2013, Shin and Ivry, 2002, Ullen and Bengtsson, 2003). Temporal learning in this type of task is said to be implicit, or ‘incidental’ (as we will refer to it). Incidental learning occurs as a by-product of non-temporal task goals, when stimuli or motor responses adhere to a strict temporal framework (see Coull and Nobre, 2008, for the proposed distinction between implicit and explicit timing). In such a situation, participants are not asked to time or recall the different intervals used in the task, but the temporal structure influences performance measures. Such incidental learning can be shown in the pattern of reaction times, which decrease over time in a manner consistently related to the temporal structure inherent to the task. SRT tasks containing a recurring sequence of temporal intervals show that learning of temporal sequences affect behavioural measures like reaction times, at least when they are combined with a stable ordinal sequence. However, it is not yet clear how the influence of learned temporal structure on behaviour and neural processing develops over time. From research on temporal orienting following explicit temporal cues, we know that explicit temporal cueing is most effective at short, compared to long intervals (Correa et al., 2006, Coull and Nobre, 1998, Miniussi et al., 1999, Nobre, 2010, Rohenkohl et al., 2014). This can elegantly be explained by the notion that, when an event has not yet occurred, the probability that it will still occur increases with time (also known as the hazard rate). Whereas at short intervals participants will be most engaged following short cues, at long intervals their engagement will have become largely independent of the cue, because once the early interval has passed, it is certain that the stimulus will thus occur late (making the cue information redundant). In other words, for events that are due to happen, knowledge about their expected timing will be most beneficial at early intervals. Based on this literature on temporal orienting following explicit cues, we hypothesise that incidentally learned temporal structure will also have the strongest impact on performance for targets that occur following short (as opposed to longer) intervals (with intervals referring to the intervals between the targets that comprise the sequence).

In the current study we used an adapted version of the SRT task used by O’Reilly et al. (2008) that used blocks containing learned and pseudorandom sequences. O’Reilly and colleagues exposed participants to blocks of trials that had a repeating ordinal sequence, a repeated temporal sequence, or both. In this study having predictable temporal information greatly facilitated learning of the ordinal sequence, but temporal information was not learned when presented in isolation (see also Buchner and Steffens, 2001, Gobel et al., 2011, Sanchez et al., 2015, Shin and Ivry, 2002). However, because our study was part of a larger magnetoencephalography (MEG) and functional magnetic resonance imaging (fMRI) investigation, several changes were made with respect to the O’Reilly et al. (2008) task, of which we now highlight the most important ones. We only used conditions where either both temporal and ordinal information were repeated, or where both types of information were changed. We used longer intervals between events to ensure reliable hazard rate effects, and used intervals between responses and stimuli (response-to-stimulus intervals; RSIs) as opposed to between stimuli intervals (stimulus onset asynchronies; SOAs) in order to strictly separate responses and stimuli in time. Shin and Ivry (2002) have reported comparable learning effects for SOA and RSI manipulations in a spatial-temporal RT task. Finally, we used new sequences, instead of pseudorandom sequences for the probe blocks, to ensure comparable second-order conditional probabilities between blocks (see Reed and Johnson, 1994). Reed and Johnson showed that it is important to keep a number of parameters the same between repeated and probe sequences. These parameters are (A) location frequency: how often each location occurs within the sequence; (B) transition frequency, how often each possible transition between locations occurs; (C) reversal frequency: how often back and forth movements occur (e.g. Position 1 – Position 2 – Position 1, see also Vaquero et al., 2006); (D) rate of full coverage: how many targets occur before each location has at least occurred once; (E) rate of complete transition usage: average number of targets before each possible location transition has occurred at least once. In addition to these constraints, we ensured that each of the three RSIs occurred once with every location, with the same RSI never occurring twice in a row.

The main goal of the current report was to evaluate the hypothesis that incidental sequential temporal orienting effects (like more explicit temporal orienting effects) are most effective at short intervals. Moreover, given our experimental set-up, we are able to address two additional points. First of all, we aimed to replicate and extend the results found by O’Reilly et al. (2008) by establishing a spatial-temporal SRT task that can be used flexibly in a behavioural setting, as well as in neuroimaging settings. We therefore optimised our task parameters for neuroimaging analysis, that benefits from sufficiently long temporal intervals and a strict separation between responses and subsequent stimuli, by virtue of the use of RSIs instead of SOAs. Second, since this study contained three different sessions, taking place over the course of two weeks, this study allows us to look at whether (and, if so, how) these incidental learning effects change with time.

## 2 Methods

### 2.1 Participants

Twenty-one young, healthy volunteers (aged 24.7 ± 3.9 (SD), 9 males) participated in this study. All were right handed according to self-report and all had normal or corrected-to-normal vision. All participants gave informed consent and the study was approved by the Central University Research Ethics Committee of the University of Oxford (MSD-IDREC-C2-2014-036). Participants received money for their participation (£10 per hour). Each participant completed three experimental sessions. The first session consisted of just the behavioural experiment; the second session, one or two days later, and the third session, taking place within two weeks subsequently, included a magnetoencephalography (MEG) and a functional magnetic resonance imaging (fMRI) scan. This manuscript will focus on the behavioural results from each of these three sessions. One participant was excluded from the main analysis because of extreme fatigue during all three experimental sessions, causing a high number of mistakes and long gaps where the sequence was interrupted, especially during the second session (percentage correct was smaller than the mean minus 3 times the standard deviation across participants). Another participant was excluded because of a very slow mean reaction time (larger than the mean plus 3 times the standard deviation across participants). Results of nineteen participants (aged 22.6 ± 4.0 (SD), 9 males) were therefore included in the final analysis, on which we here report.

### 2.2 Apparatus

The sessions were all run in rooms with similar (normal) illumination. Stimuli were created with MATLAB (The MathWorks, Inc., Natick, MA) and presented using Psychtoolbox version 3.0 (Kleiner et al., 2007). During the first session stimuli were displayed on a 24-in. Viewsonic display with a spatial resolution of 1280 × 720 pixels and a refresh rate of 60 Hz at a viewing distance of 80 cm. Responses were collected via the computer keyboard, using the ‘A’, ‘D’, ‘J’ and ‘L’ keys.

Data collected during the second session were part of a whole-head MEG recording, acquired using an Elekta NeuroMag MEG System (Elekta, Stockholm, Sweden), at the Oxford Centre for Human Brain Activity. Stimuli were back projected on a 43 × 54.5 cm translucent screen placed 120 cm in front of the participant, with a spatial resolution of 1280 × 1024 and a refresh rate of 60 Hz. Eye movements were recorded with a video-based eye tracker at 500 Hz (EyeLink 100, SR Research, Ontario, Canada). A 4-button bimanual fibre-optic response device was used to collect manual responses.

Data collected during the third, final session were part of a functional magnetic resonance imaging (fMRI) session, acquired on a 3T Siemens TIM Trio scanner (Siemens AG, Erlangen, Germany) at the University of Oxford Centre for Clinical Magnetic Resonance Research. Stimuli were back projected onto a translucent screen that participants viewed through a mirror placed on the head coil. Responses were collected using a single 4-button fibre-optic response device held with both hands.

Both the short behavioural practice session preceding the final session and the assessment of explicit awareness following the final session were presented on a 15.6-in. Dell laptop with a spatial resolution of 1280 × 1024 pixels and a refresh rate of 60 Hz at a viewing distance of 60 cm. Responses were collected via the laptop keyboard, using the ‘A, ‘D’, ‘J’ and ‘L’ keys.

### 2.3 Experimental procedure and stimuli

**Fig. 1:**
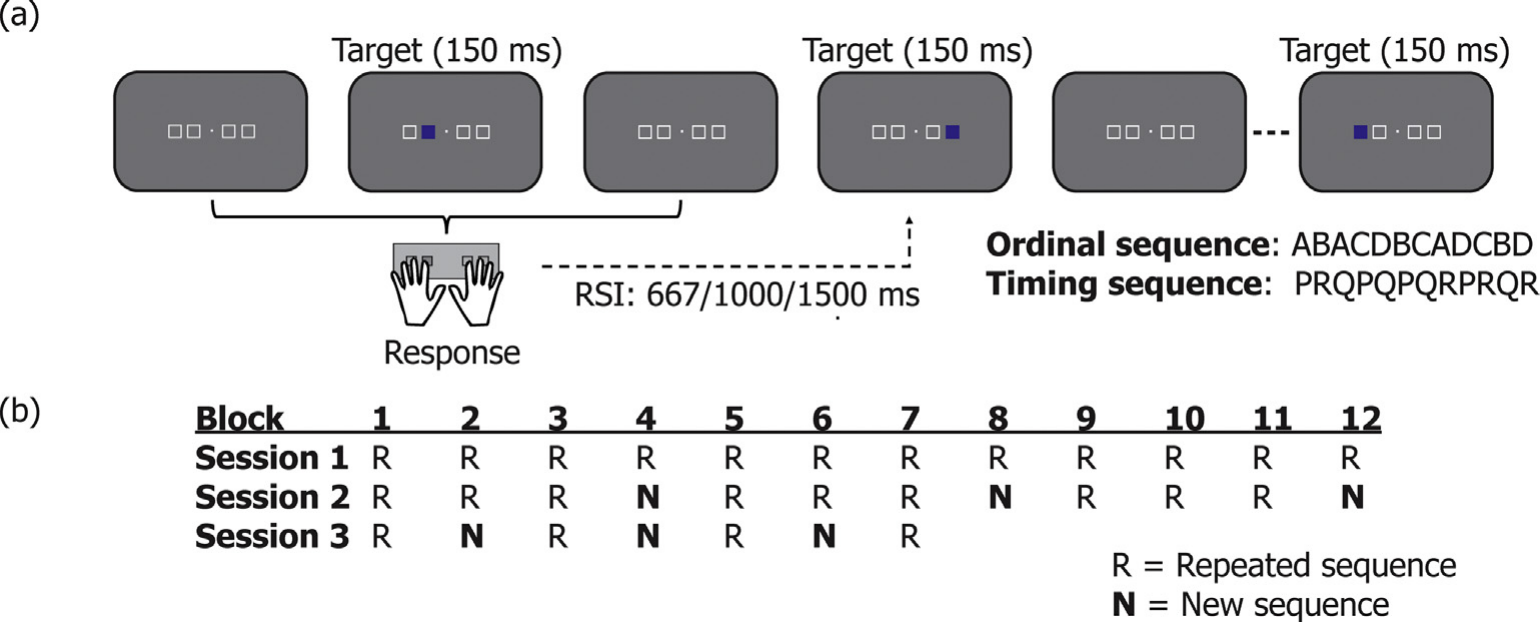
(a) Targets were presented in blue on a grey background. Four possible target locations on the screen corresponded to four button locations, to be pressed with the left and right middle and index fingers. Whenever a target appeared participants had to press the corresponding button. Unknown to participants, the order of the targets followed a repeating twelve-element cycle. The response-to-stimulus interval (RSI) preceding each target was one of three possible lengths: 667, 1000 or 1500 ms. In addition to the ordinal sequence, the order of RSIs used in this study also followed a repeating twelve-element cycle. (b) The experiment consisted of three different experimental sessions. The first session consisted of twelve repeated sequence (R) blocks. The second session contained nine R blocks and three new sequence (N) blocks in which a new spatial-temporal sequence was presented. R and N blocks alternated in the third, and final, session, for a total of four R and three N blocks. Each block within a session contained eight repetitions of either the standard or new spatial-temporal sequence.

Participants performed a modified version of a serial reaction time (SRT) task (see Fig.1a). Four locations on the screen were permanently indicated by white square outlines (2.12° × 2.12° of visual angle for each square, total width of all stimuli: 11.66° × 2.12° of visual angle) to the left and right of a small fixation square (0.17° × 0.17°of visual angle) against a grey background. Within these outlines, series of targets were presented in blue. Participants had to follow the order of targets by pressing corresponding keys on the keyboard. Participants used the middle and index finger of the left and right hand to respond in a corresponding layout to the four designated buttons of the response device (see Section 2.2).

Unknown to participants, the positions of the targets (and therefore the pattern of responses) followed a twelve-element cycle: ABACDBCADCBD, where A was the first position/left middle finger; B the second position/left index finger, C the third position/right index finger and D the fourth position/right middle finger. The next target was only displayed after a correct button press was made, i.e. participants had to correct themselves whenever they made a mistake. The response-to-stimulus interval (RSI) was either 667, 1000 or 1500 ms. The RSI factors were related by a scalar factor of 1.5. Like the ordinal responses, the RSIs followed a repeating twelve-element cycle: PRQPQPQRPRQR. The ordinal and temporal sequences were thus only linked at the level of the twelve-element cycle. Each eight repetitions of the twelve-element sequences constituted one block of trials used for analysis.

The first session consisted of twelve repeated (R) blocks (see Fig.1b). Each block consisted of 96 trials, and contained an equal number of trials of each of the possible combinations between interval and target position (and therefore response). The total number of trials in this session was 1152. Self-paced brakes were inserted every three blocks. The aim of this first session was to train participants on the task and on the spatial-temporal sequences. This session lasted approximately 30 min.

In the second session, three blocks containing the repeated sequence (R blocks) were alternated with probe blocks containing a new, unlearned sequence (N blocks), for a total of twelve blocks, i.e. the experiment consisted of nine R and three N blocks (see Fig.1b). The new sequence was repeated eight times within a N block, but a different sequence was used for each N block. Unlearned sequences were used instead of pseudorandom sequences, which are often used in SRT tasks, to ensure that sequence characteristics would be as similar as possible between R and N blocks, with the only difference being whether participants previously had been exposed to the sequence or not. Since the data collected in this session were part of a larger MEG investigation, short eight-second blinking breaks were inserted every 32 trials, with a longer break occurring after every four blocks. This second session contained a total of 864 R and 288 N trials and lasted approximately 45 min. Because the first two trials after each break were excluded from the analysis (see Section 2.4), each block started at a different (random) point within the repeated sequence, to ensure that approximately equal numbers of trials were excluded for each interval-position combination.

Before the third session one to two weeks later, participants completed a short behavioural practice session containing three R blocks interleaved by two self-paced breaks, to refamiliarise themselves with the task. The task design in the third session was adapted for fMRI; four R blocks alternated with three N blocks (see Fig.1b). After every 24 trials, there was a 16-s break, to allow the haemodynamic response to go back to baseline. The session length was approximately 25 min. The full session lasted around 60 min, including practice and assessment of explicit awareness. Similar to the second session, each block started at a different (random) point within the repeated sequence.

After the final session, we assessed explicit awareness of both the ordinal and temporal features of the task. Participants were first asked verbally about their awareness of the repeated ordinal sequence: ‘*Did you notice there was a repeated sequence?*’, ‘*How confident, on a scale from 1*–*5, would you be that you could type out the sequence?*’. After answering these two questions participants were encouraged to give this a try. Participants were presented with four empty squares, and asked to type out a sequence with a length of twelve, starting at any location, with targets showing up immediately when buttons were pressed. Subsequently, participants were asked about the temporal features of the task: *‘Did you notice anything about the timings in the experiment?’ ‘How confident, on a scale from 1*–*5, would you be that you could type out the sequence, also taking the temporal information into account?’* After answering these questions participants were asked to type out the remembered sequence for a second time, this time also taking the temporal information into account, as they remembered it. As each combination of three subsequent targets (i.e. each triplet) presented in the repeated sequence was unique, in a final assessment we presented participants with twelve combinations of two targets, as they appeared in the task, and asked them to press a button for the target they predicted to appear next. These twelve combinations were presented in random order.

### 2.4 Behavioural analysis

Behavioural data were analysed using MATLAB (The MathWorks, Inc., Natick, MA) and statistics were performed in SPSS version 22 (IBM Corp. Armonk, NY). Since only combinations of three or more stimuli (triplets) were unique and allowed for preparation for the next, upcoming stimulus, the first two trials after each break were excluded from the analysis. In addition, trials with an RT shorter and larger than 3 times the mean plus or minus the standard deviation of the participant’s mean reaction time in an experimental session were discarded. On average, 1.1 ± 0.5% (SD) of trials were rejected based on reaction times, with the maximum being 2.2% for one participant. Only correct responses were included in all RT analyses. When anticipatory responses (occurring during the RSI interval before target presentation) were made with the correct button, we included them in the RT analysis because excluding them would artificially mask the learning effect (however, our pattern of results does not change when excluding premature responses). Premature responses occurred in 0.5 ± 1.1% (SD) of trials, with the maximum being 5% of trials for one participant.

Analysis of the assessment of awareness of order was performed by dividing both typed-out sequences into 12 triplets, and calculating how many of these triplets also occurred in the repeated sequence. Furthermore, we determined how many of the twelve combinations of two targets shown in the final task were finished correctly. These numbers were statistically tested against chance. Analysis of the assessment of awareness of temporal information was performed for both typed out sequences separately by marking the four shortest RTs within a produced sequence as ‘short interval’, the four medium RTs as ‘medium interval’ and the four longest RTs as ‘long interval’. This produced sequence of short, medium and long intervals was compared against the real sequence of short, medium and long intervals to establish, per participant, the longest temporal sequence that corresponded to the repeated sequence of short, medium and long RSIs used in the task. The same comparison was done with the three sequences that appeared in the new sequence blocks; the number corresponding to the maximum overlap with the repeated sequence was statistically compared against the average maximum overlap with the three new sequence blocks.

## 3 Results

### 3.1 Temporal-ordinal learning effects

**Fig. 2:**
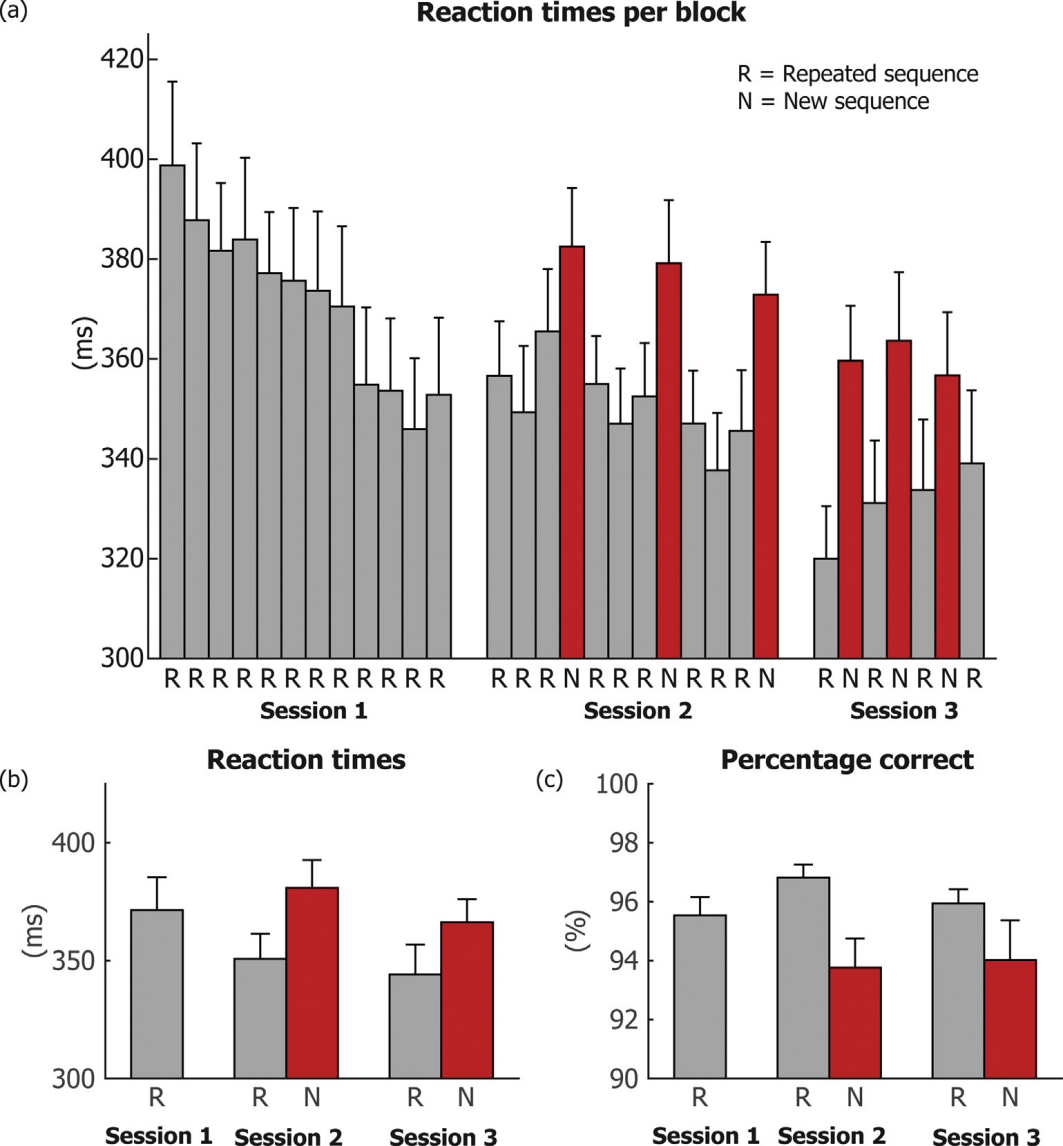
Behavioural results. Results are shown for (a/b) reaction times (RTs) and (c) percentage of correct trials (PCs) for the first, second and third sessions. Results for blocks with a repeated sequence (R) are shown in grey, while results for blocks where a new sequence (N) was presented are shown in red. Error bars present standard error of means (SEM), calculated using the variance across participants.

Since our task involved speeded responses to clearly visible targets, our main dependent variable of interest was reaction time (RT). However, for completeness, we also report results for percentage correct (PC). RTs for all three sessions are shown in Fig.2a and b. Learning in SRT tasks is generally shown by a decrease in RTs over the course of the experimental session. We expected that RTs would decrease most strongly during the first session, since this session only contained blocks with the repeated sequence, without interference from any new-sequence blocks. A repeated-measures analysis of variance (ANOVA) was performed with the mean RTs during block 1–4, block 5–8, and block 9–12 of the first session as the within-subjects variable. There was a main effect of block: F(2,17) = 17.388, p ≤ 0.0001. RTs non-significantly decreased from block 1–4 to block 5–8 (M ± SE = 388 ± 15 ms vs 374 ± 14 ms; t(18) = 1.935, p = 0.069), and significantly decreased from block 5–8 to block 9–12 (M ± SE = 374 ± 14 ms vs 352 ± 15 ms; t(18) = 5.469, p < 0.0001). A similar ANOVA was performed for the mean PC during block 1–4, block 5–8 and block 9–12, which also showed a main effect of block: F(2,17) = 4.276, p = 0.031), caused by a slightly larger PC for block 1–4 compared to block 5–8 (M ± SE = 96.0 ± 0.6% vs 95.3 ± 0.7%; t(18) = 2.77, p = 0.013), with no significant difference between block 5–8 and block 9–12 (M ± SE = 95.3 ± 0.7% vs 95.3 ± 0.6%; t(18) = −0.12, p = 0.903).

Since it was possible that RTs would continue to decrease over the course of the next two sessions, a repeated measures ANOVA was performed on the average RTs in repeated sequence (R) blocks during the first, second and third session (see Fig.2b), which indeed showed a main effect of session: F(2,17) = 8.77, p = 0.002. Subsequent paired *t*-tests showed that RTs decreased significantly both from the first to the second session (M ± SE = 371 ± 14 vs M ± SE = 350 ± 11; t(18) = 3.21, p = 0.005), and from the second to the third session (M ± SE = 350 ± 11 vs M ± SE = 329 ± 13; t(18) = 3.55, p = 0.002). A similar ANOVA was performed for PCs (see Fig.2c), showing a main effect of session: F(2,17) = 15.54, p = 0.0001. Mean performance was lower for session 1, compared to session 2 (M ± SE = 95.5 ± 0.6% vs 96.8 ± 0.4%, t(18) = 3.71, p = 0.002), but did not differ between session 2 and session 3 (96.8 ± 0.4% vs 97.2 ± 0.5%; t(18) = 0.86, p = 0.40).

A second, more convincing, way of showing sequence-specific (compared to more general) learning in SRT tasks is the increase in RT and decrease in PC when a block containing a new, (pseudo)random, sequence (denoted ‘N’) is presented instead of the learned, repeated sequence (denoted ‘R’). During both the MEG and the fMRI session, three such ‘probe blocks’ were presented (see Fig. 2). The ‘probe cost’ is the increase in RT or decrease in PC when a new, unlearned sequence is presented. Therefore, a repeated measures ANOVA was performed with the factors block type (R or N) and session (second or third). For this analysis we took separate averages across all repeated (R) and new (N) blocks within a session. This analysis confirmed a main effect of block type (F(1,18) = 29.21, p < 0.0001), and showed a main effect of session (F(1,18) = 11.76, p = 0.003), but no interaction between both variables (F(1,18) = 0.68, p = 0.419). For PC, a main effect of block type was present (F(1,18) = 16.828, p = 0.0006), but no main effect of session (F(1,18) = 0.75, p = 0.40) or interaction (F(1,18) = 0.75, p = 0.40) was found.

### 3.2 Temporal orienting effects across intervals

**Fig. 3:**
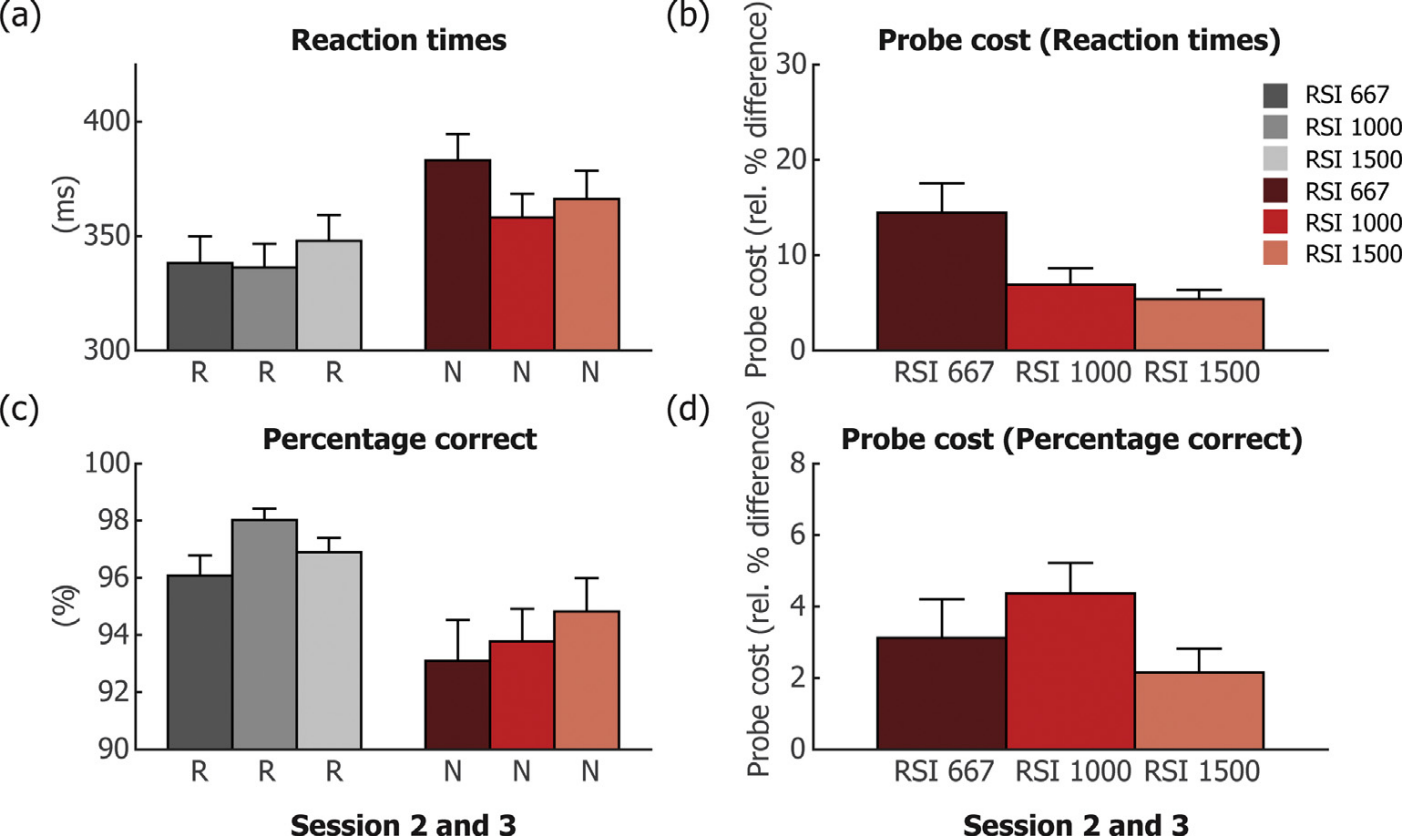
Results are shown for (a and b) RT and (c and d) PC for each of the three response-to-stimulus intervals (RSIs, 667/1000/1500 ms), separate for repeated sequence (R) and new sequence (N) blocks, averaged across all R or N blocks of the session. The probe cost, shown in (b) and (d) for RT and PC was calculated as the relative difference between the average of all repeated sequence (R) and all new sequence (N) blocks across both sessions, for each RSI length. Error bars present SEM.

Both the decrease of RTs over the course of the experiment and the increase in RTs and decrease in PC when probe blocks were presented, indicate that participants incidentally learned the repeated spatial-temporal sequence. To investigate specifically the utilisation of the learned temporal structure in the sequences, we further investigated the difference in probe costs for the different response-to-stimulus intervals (RSIs) that were used in the task (667, 1000 and 1500 ms). Fig. 3 shows the RT (a and b) and PC (c and d) results for the different RSIs.

To establish if the three RSIs indeed showed different RT patterns when comparing repeated (R) versus new (N) sequences, we performed a repeated-measures ANOVA with the factors RSI (667/1000/1500 ms), block type (R or N) and session (two or three). This analysis showed a main effect of RSI (F(2,17) = 12.23, p = 0.001), block type (F(1,18) = 40.51, p < 0.0001) and session (F(1,18) = 10.61, p = 0.004; see Fig. 2 for the main effect of session). Critically, there was also an interaction between RSI and block type (F(2,17) = 19.42, p < 0.0001), but no interaction between RSI and session (F(2,17) = 1.35, p = 0.286), no interaction between block type and session (F(1,18) = 0.457, p = 0.508) and no three way interaction (F(2,17) = 0.434, p = 0.655). This interaction between RSI and block type can be evaluated by looking at the probe cost as a function of the different RSIs. However, before we could statistically compare the probe costs for the different RSIs, we first aimed to establish that there were indeed ‘costs’ for each RSI, i.e. that the difference between new and repeated blocks was present for each of the three RSIs. We therefore performed pairwise *t*-tests between the average of all repeated sequence (R) blocks and all new sequence (N) blocks of the second and third session (collapsed across sessions because we found no three-way interaction with session; and not using the first session because it did not contain any probe blocks). Results show that there were indeed significant probe costs on RT for each of the three RSIs (short RSI: M ± SE = 337 ± 13 ms vs 383 ± 12 ms; t(18) = 5.53 p < 0.0001; medium RSI: M ± SE = 333 ± 11 ms vs 358 ± 10 ms; t(18) = 3.90 p = 0.001; long RSI: M ± SE = 347 ± 11 ms vs 366 ± 12 ms; t(18) = 5.24 p < 0.0001). We then investigated probe costs across the different RSIs (collapsed across sessions 2 and 3; see Fig.3b). A repeated-measures ANOVA showed a main effect of RSI (F(2,17) = 21.26, p < 0.0001). Pairwise *t*-tests showed that probe costs for the short RSI were larger than probe costs for the medium RSI (M ± SE = 46 ± 8 ms vs 24 ± 6 ms; t(18) = 6.53, p < 0.0001) and also larger for the short RSI than for the long RSI (M ± SE = 46 ± 8 ms vs 18 ± 3 ms; t(18) = 3.76, p = 0.001, but did not differ between the medium and long RSI (M ± SE = 24 ± 6 ms vs 18 ± 3 ms; t(18) = 1.12, p = 0.276).

The same analysis was performed for PC values (see Fig.3c and d). First, we performed a repeated-measures ANOVA with the factors RSI (667/1000/1500 ms), block type (R or N) and session (two or three). This analysis showed a main effect of block type (F(1,18) = 22.839, p < 0.0001), a trend towards a main effect of RSI (F(2,17) = 3.552, p = 0.051), but no effect of session (F(1,18) = 0.361, p = 0.555). Critically, there was again an interaction between block type and RSI (F(2,17) = 8.11, p = 0.003), but no interaction between RSI and session (F(2,17) = 2.439, p = 0.117), no interaction between block type and session (F(1,18) = 0.033, p = 0.858) and no three way interaction (F(2,17) = 1.214, p = 0.321). Since there was a main effect of block type, and an interaction between block type and RSI, we again evaluated if there were significant probe costs for each of the three RSIs (collapsed across sessions, because there was no main effect of session, or interaction with session). This was indeed the case (short RSI: M ± SE = 96.08 ± 0.63% vs 93.09 ± 1.30%; t(18) = 2.90 p = 0.010; medium RSI: M ± SE = 98.01 ± 0.39% vs 93.78 ± 1.12%; t(18) = 5.20 p < 0.0001; long RSI: M ± SE = 96.89 ± 0.50% vs 94.82 ± 0.93%; t(18) = 3.23 p = 0.005). Subsequently, a repeated-measures ANOVA on probe costs showed a main effect of RSI (F(2,17) = 8.11, p = 0.003). Subsequent pairwise *t*-tests showed that for PC, probe costs were larger for the medium RSI than for the long RSI (M ± SE = 4.24 ± 0.82% vs 2.07 ± 0.65%; t(18) = 3.12, p = 0.006). But did not differ between the short and medium RSI (M ± SE = 2.99 ± 1.03% vs 4.24 ± 0.82%; t(18) = 1.55, p = 0.138) or the short and the long RSI (M ± SE = 2.99 ± 1.03% vs 2.07 ± 0.65%; t(18) = 0.75, p = 0.463).

**Fig. 4:**
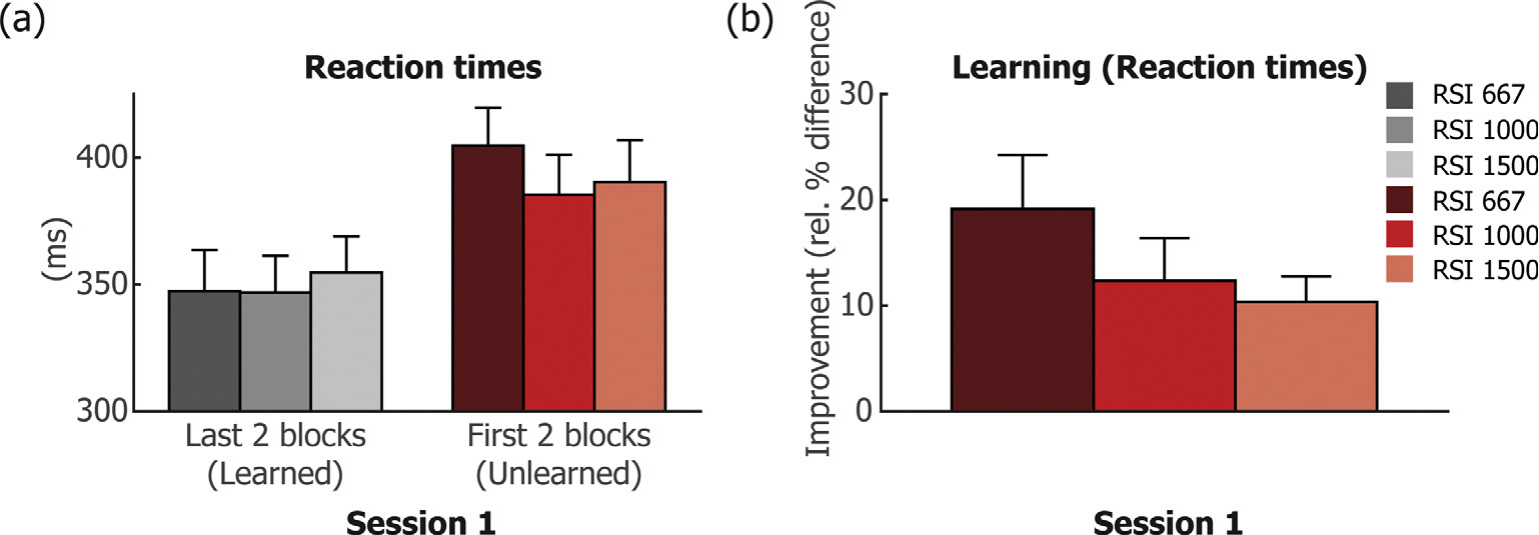
Results are shown for RTs for each of the three response-to-stimulus intervals (RSIs, 667/1000/1500 ms), separate for the first two and last two blocks of the first session. The learning effect, shown in (b) was calculated as the relative difference between the first two and the last two blocks, for each RSI length. Error bars present SEM.

After establishing a main effect of RSI when comparing new and repeated blocks, we hypothesised that it might be the case that a similar pattern of RT results could be found when comparing the first two and the last two blocks of the first session. During the first two blocks of this session, the standard sequence was still unlearned and therefore one would expect the pattern of RTs to be similar to the pattern shown in new (N) blocks in session two and three, while in the last two blocks of the first session the sequence was learned (shown by the decrease in RTs over the course of the first session), and therefore would likely be similar to the pattern showed in repeated (R) blocks in session two and three. Relevant data are depicted in Fig. 4. Before comparing the size of learning effects between the different RSIs, we first established if learning effects in the first session were indeed present for each RSI. Pairwise *t*-tests show that RTs indeed decreased significantly between the first and last two blocks of session 1 for each RSI (short RSI: M ± SE = 347 ± 16 ms vs 405 ± 15 ms; t(18) = 4.62, p = 0.0002; medium RSI: M ± SE = 347 ± 14 ms vs 385 ± 16 ms; t(18) = 3.28, p = 0.004; long RSI: M ± SE = 355 ± 14 ms vs 390 ± 16 ms; t(18) = 4.20, p = 0.001). For PC there was a difference between the first and last two blocks for the medium RSI (M ± SE = 97.78 ± 0.60% vs 95.81 ± 0.86%; t(18) = 2.52, p = 0.021) but not for the short or long RSI (short RSI: M ± SE = 95.31 ± 1.01% vs 95.81 ± 0.75%; t(18)=-0.52, p = 0.607; long RSI: M ± SE = 96.05 ± 0.70% vs 95.31 ± 1.01%; t(18) = 0.78, p = 0.447). Because RTs decreased significantly between the first two and the last two blocks of the first session for all intervals, we tested for potential differences in the size of this decrease between the different RSIs (see Fig.4b) with a repeated measures ANOVA, which showed that there was indeed a main effect of RSI (F(2,17) = 8.363 p = 0.003). The decrease in RT was larger for the short RSI than for the medium RSI (M ± SE = 57 ± 12 ms vs 39 ± 12 ms; t(18) = 4.16 p = 0.001) and larger for the short RSI than for the long RSI (M ± SE = 57 ± 12 ms vs 36 ± 9 ms; t(18) = 3.05, p = 0.007), but did not differ between the medium and long RSIs (M ± SE = 39 ± 12 ms vs 36 ± 9 ms; t(18) = 0.51, p = 0.617). This pattern of results is therefore similar to the pattern of results found for the probe costs for the different RSIs.

### 3.3 Assessment of awareness

#### 3.3.1 Assessment of awareness of ordinal sequence

After the last experimental session, participants were asked verbally if they noticed anything about the experiment. Nine participants answered that they did not notice anything in particular, while ten participants mentioned that they noticed there was a repeating pattern. After being told about the repeated sequence, fifteen participants mentioned they did indeed notice that the order sometimes repeated. However, most participants also mentioned that they had not actively tried to learn the sequence when they realised this, it was more a feeling of occasionally noticing being able to predict the next target. In line with this result, participants were not very confident they would be able to type out the sequence; when asked to rate on a scale from 1 to 5 how confident they were they could type out the sequence the average rating was 1.74 ± 0.25 (SE).

We also asked participants to generate the remembered sequence from memory (see Section 2.3). Unfortunately, results of five participants had to be excluded from the analysis of these data, because of a mistake in the stimulus presentation script that caused some responses not to be recorded. Results of fourteen participants were therefore included in this analysis. Results for the ordinal sequence showed that participants were consistent across both times they were asked to type out the sequence and on average produced 5.3 ± 0.6 (M ± SE; out of twelve) correct triplets during their first try (one-sample *t*-test against 4 expected by chance: t(13) = 2.223, p = 0.045) and 5.2 ± 0.3 correct triplets during their second try (one-sample *t*-test against 4: t(13) = 4.660, p < 0.001).

The results for the final task, including results for all nineteen participants, in which participants were presented with twelve combinations of two targets (as they appeared in the task) and had to predict the next target, showed that on average 5.3 ± 0.4 (M ± SE) triplets were finished correctly (t-test against 4 correct to be expected by chance: t(18) = 2.839, p = 0.011). Results were therefore very similar to the results for the self-generated sequences, and these results together suggest that, while participants were not very confident in their explicit knowledge of the ordinal structure of the repeated sequence, they gained at least some intuition for the ordinal aspects of the sequence, as they were able to perform the generation and prediction task slightly above chance.

Because the average performance was above chance when reproducing the sequence, we assessed if our main behavioural interaction of interest (i.e. the interaction between RSI and block type) would still hold when excluding the ten participants that initially said they noticed there might have been repeating pattern. This was indeed the case: F(2,7) = 8.81, p = 0.012). The main effects of RSI and block type were also still present (RSI: F(2,7) = 9.672, p = 0.01; block type: (F(1,8) = 13.891, p = 0.006), while there again was no two-way or three-way interaction with session (RSI and session: p = 0.787, block type and session: p = 0.947, block type, RSI and session: p = 0.306).

#### 3.3.2 Assessment of awareness of temporal sequence

After being asked about the ordinal sequence, participants were asked about the temporal features of the task. When asked about the repeating temporal sequence, seven participants mentioned that they noticed that timings were not always the same (no one, however, mentioned noticing a repeated temporal structure). Six of these seven participants were part of the ten participants that mentioned noticing there might have been a repeated pattern, see above. No one was confident they would remember the timings when typing out the sequence; only two participants rated their confidence as 2, while everyone else rated their confidence as 1 for this question (M ± SE = 1.11 ± 0.07).

Because the temporal sequence only contained three different RSIs, and therefore, unlike the ordinal sequence, did not contain unique triplets, we followed a different procedure for establishing awareness (see Section 2.4). The length of the produced sequence of short, medium and long intervals that matched with the sequence of intervals in the repeated sequence was compared against the (mean) length that matched with the three new sequences. The correctly produced sequence of short, medium and long intervals that matched with repeated (R) blocks had an average length of 2.79 ± 0.47 (M ± SE) for the first try (i.e. before they were informed about the temporal sequence) and 2.07 ± 0.27 for the second try (i.e. after they were informed about the temporal sequence). For new (N) blocks this was an average of 2.40 ± 0.29(M ± SE) for the first try (average across the scores for the three separate blocks) and 2.17 ± 0.27 for the second try. Because there was no indication that being informed about the temporal sequence made a difference to these scores (scores for the second attempt were numerically lower, rather than higher) a pairwise *t*-test was performed between the average of the first and second try for the results for R and N blocks, showing no difference in average produced length between both conditions (t(13) = 0.689, p = 0.503). This shows that, as already suggested by the low confidence ratings, participants were not able to reproduce the temporal structure of the task, and that participants thus gained no explicit knowledge of the temporal structure in the repeated sequences.

## 4 Discussion

In this study, participants performed a SRT task involving complex combined spatial-temporal visuomotor sequences. We aimed to replicate and extend previous results (O’Reilly et al., 2008), by establishing an SRT task that could be used flexibly in both behavioural, and neuroimaging settings. More importantly, we addressed the hypothesis that incidental sequential temporal orienting effects would be largest for targets following short (as opposed to longer intervals) between temporally structured sequence elements, thereby paralleling classical observations in tasks using explicit temporal cues (Correa et al., 2006, Coull and Nobre, 1998, Miniussi et al., 1999, Nobre, 2010, Rohenkohl et al., 2014). Our results show, as predicted, that participants learned the information embedded in the sequences, which is shown by RTs becoming faster over the course of especially the first session, and by slower RTs when probe blocks were presented during the second and third sessions, nicely replicating learning effects found in ‘typical’ spatial SRT studies (see Schwarb and Schumacher, 2012, for a review of the spatial SRT literature) and spatial-temporal SRT studies like the study by O’Reilly et al. (2008). Importantly, we also found that indeed RT differences between new and repeated blocks were largest for the short interval, compared to the medium and long intervals, and that this effect already developed over the course of the first session, where the repeated sequence was gradually acquired, without interruption by probe blocks.

Our task tested for incidental effects of learned temporal structure. Participants were not required to form declarative knowledge of the intervals and their sequence. Nevertheless, we were interested in assessing the degree of awareness participants may have had about the temporal parameters used. We therefore conducted a number of tests in which (parts of) the learned sequence had to be reproduced and asked participants for confidence ratings. For the ordinal sequence, confidence ratings were low, but the subsequent sequence generation tests showed that participants were able to reproduce triplets occurring in the repeated sequence (slightly) above chance. Due to low confidence ratings and the number of correctly reproduced triplets only being slightly above chance, we consider the ordinal learning effects to be largely, but potentially not completely, incidental. For the temporal aspects of the task (which were the main focus of the current manuscript), it is key to point out that (1) no one noticed that there was a repeated sequence, (2) confidence ratings were even lower than for the ordinal aspects of the task, and (3) the reproduction task showed no sign of any explicit learning. We therefore consider it very unlikely that participants had any explicit knowledge of intervals, and therefore attribute our key results to the non-deliberate influence of incidentally acquired temporal expectations that remained, at least for a large part, unavailable to conscious report.

RTs were the obvious dependent variable in this study, and showed all hypothesised effects. For completeness, we also reported percentage correct (PC), although we noted that PC effects were almost at ceiling. While some effects were also evident for PC, other effects were less reliable for this measure. PC results for some of the reported effects nicely follow the RT results: both measures show probe costs when comparing new (N) and repeated (R) blocks, and RTs decrease for R blocks across the whole experiment, while PC values increase, at least between the first and second session. However, when looking at learning effects within the first session, or at learning effects for the different RSIs, effects were less consistent between both measures. While RTs decrease over the course of the first session, PC values slightly decrease as well (where an increase would be expected with learning). For the analysis of RSIs, different patterns of results were found for RT versus PC. While for RT, the largest probe costs were clearly present for the short RSI, for PC the effect was most pronounced for the medium interval. We believe that the decrease in PC values over the course of the first session may be explained by factors that do not necessarily have to do with learning, like a speed-accuracy trade-off over the course of the session.

Although our task does not allow us to distinguish between pure temporal learning effects and spatial-temporal learning effects that only apply when both sequences are combined, O’Reilly et al. (2008) did make such a distinction, by also including a condition in which the temporal sequence was repeated, while the ordinal sequence was random. While they did not find any evidence of learned temporal sequence, without a correlated (repeated) ordinal structure, having a fixed spatial-temporal structure was beneficial, compared to a condition with only a fixed ordinal structure and random timing. These results are similar to other temporal sequential learning studies finding no evidence of the learning of a temporal sequence, in the presence of a random or uncorrelated ordinal/motor sequence (Buchner and Steffens, 2001, Gobel et al., 2011, Sanchez et al., 2015, Shin and Ivry, 2002; however, also see Brandon et al., 2012, Karabanov and Ullen, 2008, Salidis, 2001, Schultz et al., 2013, Ullen and Bengtsson, 2003). An interesting proposal by Kornysheva et al. (2013, but see also O’Reilly et al., 2008) holds that an independent representation of a learned temporal sequence can only be revealed when this temporal sequence is recombined with a new, stable ordinal sequence. Since there were some dissimilarities between the task used by Kornysheva et al. and our task (the use of inter-stimulus-intervals instead of RSIs and the use of sequences with only 5 stimuli instead of 12, like we used), it would be interesting to establish in future research whether these predictions hold for longer, more complex sequences, when there is less explicit awareness.

In addition to replicating previous results, we set out to investigate how the influence of learned temporal structure develops over time. We addressed the hypothesis that the influence of learned temporal sequence would be largest for targets following short RSIs, similar to effects found for explicit forms of temporal cueing at short, compared to long intervals (see the initial study by Coull and Nobre, 1998; but also e.g. Correa et al., 2006, Correa and Nobre, 2008, Miniussi et al., 1999, Nobre, 2010, Rohenkohl et al., 2014). These studies consistently show that temporal orienting of attention induced by temporal cues is most effective at short, compared to long intervals, due to evolving hazard rates, i.e. the increase in probability over time that an event will occur, given that it has not occurred yet. Because of such hazard rate effects, RTs for targets following (uncued) short intervals tend to be larger than RTs for targets following longer intervals. However, when the short interval is cued, engagement will be larger much earlier in the interval, giving a large performance benefit. For targets following long intervals the cue has less influence on behaviour, since once the early interval has passed, it is certain that the target will occur late (see Nobre and Heideman, 2015 for a review on hazard rates, temporal cues and temporal expectations induced by other types of temporal bias). Our results show that incidentally learned temporal structure (induced by the repeated temporal sequence) can have a similar influence on RTs. In our task, temporal orienting induced by the learned temporal structure clearly was most beneficial for targets following short, as opposed to longer RSIs, similar to the explicit cueing effect described above. One difference with results generally found in cued temporal orienting studies is that we did also find a repeated versus new benefit for the longest interval, while such a benefit is usually not found with explicit cues, at least when the target is certain to appear (Chauvin et al., 2016, Correa et al., 2006). However, it is feasible that this ‘residual benefit’ at the longest interval may be fully carried by the ordinal sequence structure (rather than the temporal sequence structure), given that repeated and new sequences differed in both dimensions. In fact, we cannot exclude the possibility that all results are due to pure ordinal expectations. However, we consider this unlikely because O’Reilly et al. (2008) showed substantial additional benefit of temporal structure when it was combined with ordinal information. Furthermore, our results show characteristic behavioural effects of temporal expectations being strongest at short intervals. In future studies one could aim to decouple the ordinal and temporal sequential predictions, to be able to distinguish purely temporal and purely ordinal predictions. It would also be interesting to investigate whether effects of cued temporal orienting and the effects of incidentally learned temporal order rely on similar neural mechanisms, or are different in mechanistic terms.

Two previous SRT studies also investigated the influence of learned temporal structure. Salidis (2001) used a purely temporal SRT task (i.e. without the correlated spatial sequence) to investigate incidental temporal sequence learning in a between-subjects design using three different response-to-stimulus intervals. One single response button was used to respond to either each tone**.** Tones were presented in repeated sequences of temporal intervals for six beeps, or presented in a random order of intervals. This study showed a main effect of RSI, but contrary to our results, RTs decreased with increasing RSI length, even for the group in the repeated sequence condition. However, there are some important differences with the current study. The shortest RSI that Salidis used was 180 ms, while the shortest RSI in our task was 667 ms, allowing for longer preparation, which might explain the discrepancy in results. Furthermore, in the Salidis (2001) task the upcoming ordinal stimulus was always fully predictable, even when the timing was perturbed, while in our case four different targets (and thus responses) were used. Still, also in this study, the largest probe costs were found for the short RSI (compared to the long and medium RSIs) when perturbing the temporal sequence, and the RT difference between the sequenced and random group decreased with RSI length. Another study also testing for the effect of learned temporal structure (Shin and Ivry, 2003) did not find a systematic relationship between RSI and learning. Again, however, there were some important differences with the current study. First of all, Shin and Ivry focused on the influence of RSI on *spatial* learning, i.e. they compared spatial-temporal blocks, to blocks in which the spatial sequence was changed, but the sequence of RSIs remained unchanged. Although the study showed no signs of a learned temporal sequence, in the absence of a correlated ordinal sequence, it is possible that keeping the same temporal structure influenced the results. Furthermore, the length and range of RSIs used in this study was smaller (200, 500 and 800 ms) than used in the current study, making the task less sensitive to hazard rate effects (and therefore to the influence of temporal orienting). Finally, the participants included small groups of only 8–10 patients and elderly participants, who are generally more variable in their reaction times (Fozard et al., 1994).

Since our study contained three different sessions, taking place over the course of two weeks, another goal of our study was to look at whether and how the incidental temporal sequential learning effects change with time. Although we found that RTs continued to decrease over the course off the whole experiment (RTs for the third session were faster than for the second session), the pattern of RT results for the different RSIs developed over the course of the first session and then remained stable for the rest of the experiment. Therefore, once the temporal structure was incidentally acquired it could be applied consistently, and it continued to improve performance at a similar level (particularly at the short interval) throughout the rest of the experiment.

In conclusion, our results suggest that incidentally acquired spatial-temporal expectations have a robust influence on visually guided behaviour, similarly to what happens following explicit temporal cues, the greatest benefits occur after short, compared to long inter-element intervals.
